# Supplementary material for: The interactome of intact mitochondria by cross-linking mass spectrometry provides evidence for coexisting respiratory supercomplexes
Source: Mol Cell Proteomics. 2017 Dec 8;17(2):216–32. doi: 10.1074/mcp.RA117.000470 (PMC5795388; doi:10.1074/mcp.RA117.000470)
Supplement: Supplemental Data [file supp_RA117.000470_133922_0_supp_23978_fzffwf.zip › spectra_annotation/mito_spectra_annotation/282-1-21-1-15-1.pdf]

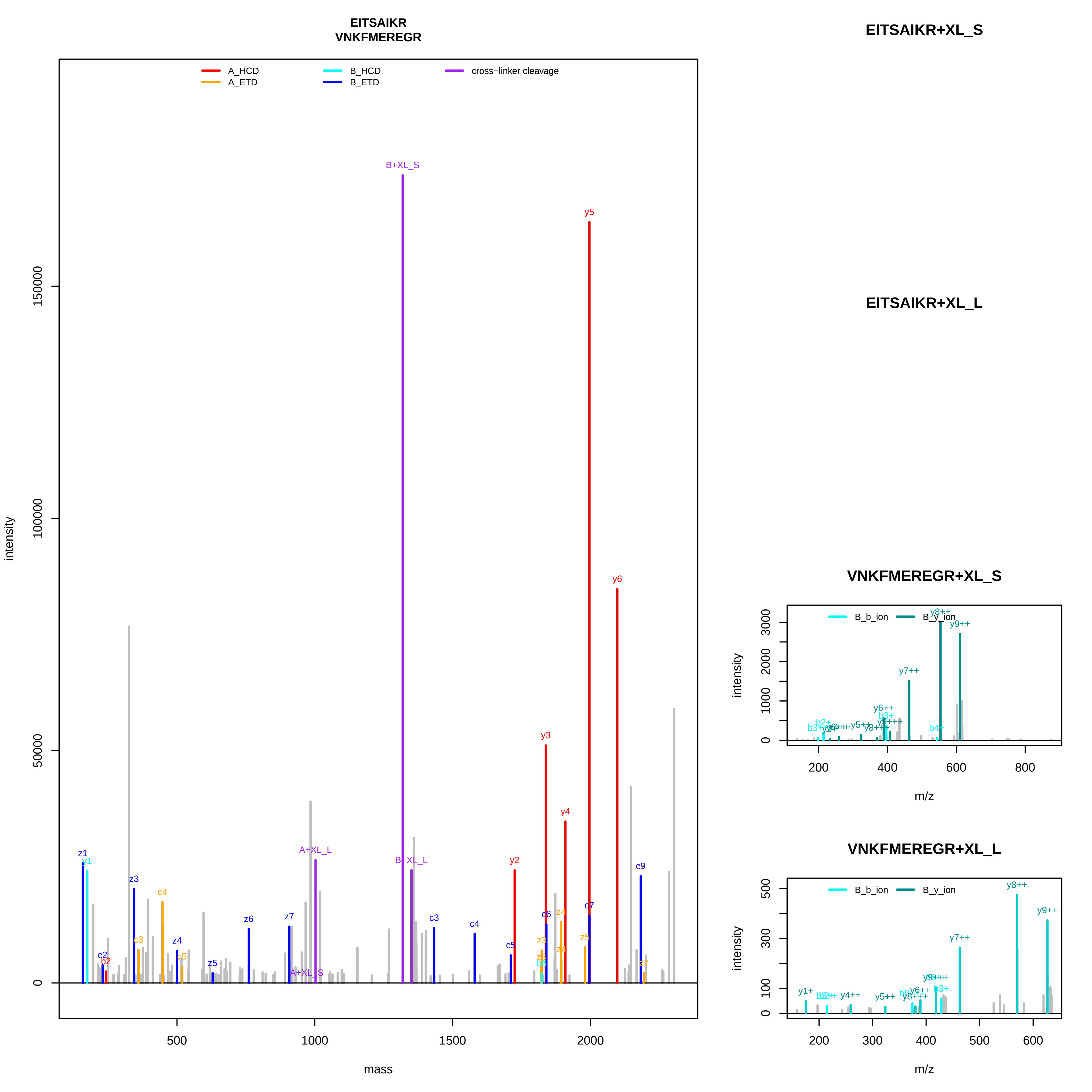

EITSAIKR
VNKFMEREGR
A_HCD
A_ETD
B_HCD
B_ETD
cross−linker cleavage
intensity
mass
0
50000
100000
150000
500
1000
1500
2000
B+XL_S
y5
y6
y3
y4
y2
A+XL_L
B+XL_L
A+XL_S
z1
y1
c2
b2
z3
c4
c3
z4
c5
z5
z6
z7
c3
c4
c5
c6
c7
c9
z7
EITSAIKR+XL_S
EITSAIKR+XL_L
VNKFMEREGR+XL_S
B_b_ion
B_y_ion
y8++
y9++
y7++
y6++
intensity
m/z
0
1000
2000
3000
200
400
600
800
VNKFMEREGR+XL_L
B_b_ion
B_y_ion
y8++
y9++
y7++
y1+
y4++
y5++
intensity
m/z
0
100
300
500
200
300
400
500
600
